# Gynecologic manifestations in Emberger syndrome

**DOI:** 10.4274/tjod.galenos.2021.53050

**Published:** 2021-03-12

**Authors:** Hasan Yüksel, Emre Zafer

**Affiliations:** 1 Aydın Adnan Menderes University Faculty of Medicine, Department of Obstetrics and Gynecology, Division of Gynecological Oncology, Aydın, Turkey; 2 Aydın Adnan Menderes University Faculty of Medicine, Department of Obstetrics and Gynecology, Specialty in Medical Genetics, Aydın, Turkey

**Keywords:** Emberger syndrome, GATA2, HPV, immunodeficiency, vulvar cancer

## Abstract

Immune system vulnerability seems to play a significant role in the development and malignant transformation of pre-malignant squamous cell lesions. Emberger syndrome is a condition that affects the immune system, which is caused by GATA2 gene mutations. Our objective was to present the gynecologic expressions of this rare syndrome in our case. Here, we discussed a relatively young patient with findings related to Emberger syndrome such as recurrent infections, myelodysplastic syndrome, lower extremity edema, and multifocal, multicentric premalignant/malignant genital lesions. Sequencing of the GATA2 gene was accomplished for suspected Emberger syndrome and a point mutation in intron5, c1143+8C >T was detected. Gynecologists may play an important role in the early detection of Emberger syndrome and guiding multidisciplinary treatment options as the initial signs related to this rare entity can appear on the genitalia.

## Introduction

Emberger syndrome is an autosomal dominant condition with incomplete penetrance and is caused by mutations in GATA-binding protein 2 (*GATA2*) gene^(1,2,3)^. GATA2 is located on the long arm of chromosome 3 (3q21). Its protein product belongs to a family of transcription factors known for having a role in the differentiation and proliferation of hematopoietic cell progenitors.

Familial aggregation and sporadic occurrences have both been reported^(1,4)^. GATA2 protein deficiency can lead to lymphopenia, and monocytopenia resulting in a tendency to viral, mycobacterial, and fungal infections^(5)^. Myelodysplastic syndrome (MDS) and acute myeloid leukemia (AML) are among the common presentations of Emberger syndrome. Along with immunodeficiency, hematologic abnormalities and lower extremity lymphedema, some other physical features such as sensorineural deafness, hypotelorism, webbed neck, and condylomas may co-occur^(1,3,4,6)^.

The predisposition to certain infections due to immune and hematologic system abnormalities observed in this syndrome can be difficult to manage. Human papillomavirus (HPV)-related lesions may cause serious problems, especially in young patients with prominent immune deficiencies^(4)^. Clinically challenging disseminated genital condylomas have been reported. Although squamous cancer of the vulva is rarely encountered under the age of 40 years, patients with Emberger syndrome seem to tend to develop such neoplasms caused by HPV^(7,8)^.

However, HPV-related premalignant or malignant diseases in patients with GATA2 mutations have been rarely discussed in gynecologic oncology literature. A few available reports have focused mostly on its hematologic representation. In this text, we presented a case of Emberger syndrome with multifocal/multicentric HPV-related premalignant and malignant genital lesions. Early diagnosis was emphasized to slow down disease progression.

## Case Reports

A 41-year-old G1P1 woman was referred to our outpatient clinic with a history of an “abnormal smear result,” which was dated more than a decade ago. She also had a history of occasional blood transfusions for chronic and severe anemia in the internal medicine department. Cervical biopsies were performed, and results were consistent with cervical carcinoma *in situ*. The patient had opted for a hysterectomy due to her social and health insurance problems. A total abdominal hysterectomy was performed in 2008 and the final pathology was reported as carcinoma *in situ* of the cervix. She continued to need blood transfusions twice a year for anemia during her follow-up. Also, she was followed up for chronic hepatitis B.

After hysterectomy, the patient had vaginal smears at irregular intervals, which were reported as benign/low-grade results. Over the years, she had treatment for various infections, mostly of upper respiratory origin. Once, she had been hospitalized for pericardial tamponade of undetermined etiology in 2014. Subsequently, the patient was diagnosed as having vulvar squamous cell cancer and a radical vulvectomy was performed. The final pathology was vulvar squamous cancer (pT1b), positive surgical margins with vulvar intra-epithelial neoplasia (VIN) grade 3. The specimen was positive for HPV immunohistochemistry. Three months after the surgery, one skinning re-excision was performed for positive margins, which were later confirmed as VIN2+ lesions.

An investigation for the cause of anemia and thrombocytopenia revealed splenomegaly, a year after her last surgery. Subsequently, the patient had intractable hematuria, which led to a bone marrow biopsy resulting in a diagnosis of AML. The patient underwent chemotherapy without any complications and MDS developed in the following years.

Recently, she developed new VIN and anal intraepithelial neoplasia of various grades on perineal biopsies and a wide local excision was performed. The final pathology report revealed focal invasive squamous cell cancers with clear margins. Her last vaginal smear was low-grade squamous intraepithelial lesion, and was positive for HPV type 89. Meanwhile, the patient was treated with antiviral treatment for hepatitis B.

**Figure 1:**
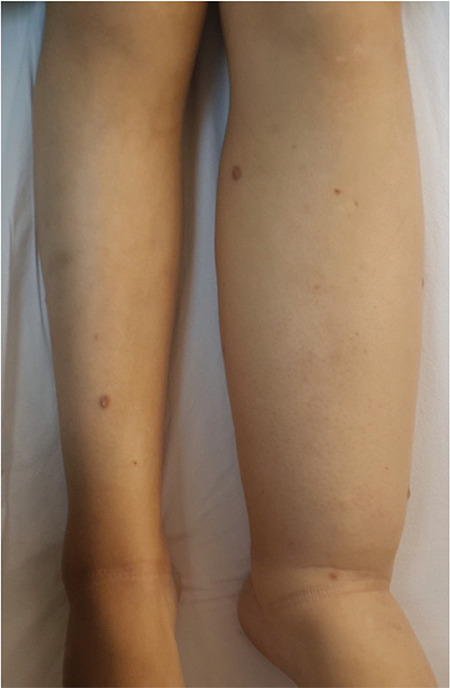
Lymphedema of the left lower extremity

On physical examination, there was left lower extremity lymphedema, which she recalled having since her childhood (Figure 1). There were also brown macules, mainly on the edematous leg.

With many matching clinical findings, Emberger syndrome was suspected. Sequencing was accomplished for the *GATA2* gene and a point mutation in intron5, c1143+8C >T was detected.

Later, the patient was hospitalized at another hospital for pneumonia and she died of myocardial infarction. Her family history was negative among her five siblings and parents for similar conditions.

## Discussion

When the etiology of genital premalignant and malignant squamous lesions is questioned, we usually refer to the term of “immune deficiency” among many factors. However, this etiologic subtitle is not scrutinized except in few situations such as in immune suppressant use and HIV infection. Current knowledge is expanding with ever-advancing research and technology. Both *in vitro* and clinical genetic studies contribute to the recognition, classification, and treatment of diseases as in immune deficiencies. For instance, *CXCR4*, *DOCK8, EVER1, EVER2 *and* GATA2* gene mutations are found to be linked to HPV infection susceptibility^(9)^. Therefore, in patients with such disseminated and severe findings, or with persistent, and recurrent lesions, early recognition of a possible immune deficiency and/or genetic syndrome can be important. Indeed, there is a need for clear algorithms to guide gynecologists as to when and where to consider such diagnoses and run these tests.

Several mutations affecting the gene function have been reported in Emberger syndrome. Also, several different phenotypes of Emberger syndrome have been described among both familial and sporadic cases^(1,10)^.

Susceptibility to HPV-related lesions and nontuberculous mycobacterial infections is common in Emberger syndrome. Ebstein-Barr virus-related cancers may also be encountered^(4,8)^.

MDS is a clonal stem cell disease, which is a common finding in patients with Emberger syndrome. It may present with chronic mild cytopenia or it can cause symptomatic anemia, infection, and bleeding. Progression to AML can be a serious problem. In addition to the predisposition to MDS/AML, some other features such as pulmonary alveolar proteinosis, congenital lymphedema, and sensorineural hearing loss can also be observed.

According to a study of a small group of 57 (31 women, 26 men) patients with GATA2 mutations, 82% of patients had infection-related presenting symptoms^(4)^. HPV was first among these infections: genital warts in 55%, genital dysplasias in 48% (vulvar, cervical, vaginal and anal), and cervical squamous cell cancers in 6% was reported in female patients. HPV-related head and neck cancers have also been reported.

It is difficult to give the exact prevalence of Emberger syndrome. To our knowledge, this is the first case to be reported from Turkey. The patient’s slides revealed typical koilocytic changes and positive HPV staining and HPV type 89 was demonstrated via polymerase chain reaction in the last vaginal smear.

The patient had no family history of a similar phenotype; therefore, the case was accepted as sporadic. Sequencing and analysis of the *GATA2* gene identified an alteration in intron5, c1143+8C >T. A wide range of mutations and even whole-gene deletion can lead to the wide spectrum of clinical findings described in this syndrome. For instance, viral infections and lymphedema were reported to be more common in individuals with null mutations^(4)^. In a genome-wide study on endometriosis, GATA2 was found to be suppressed by hypermethylation in endometriotic cells but was normal in stromal cells^(11)^. This is an interesting point to keep in mind that, even though a certain gene’s sequence looks intact and not mutated, that gene can still be “silenced” by certain mechanisms, mostly by hypermethylation.

Disease-causing alterations, namely mutations and pathogenic variants, are usually present in exons, the coding regions. However, there have been reports on mutations in introns, in enhancer elements, affecting the transcription process and causing Emberger syndrome^(12)^. In our patient, there were no coding region mutations. The only sequence change was found in the intron region.

Genotype-phenotype relations in rare syndromes are established better as more cases are reported, and as more variants/mutations are characterized. Even though this variant has not been reported before, the patient’s clinical presentation strongly suggests the presence of such a genotype-phenotype correlation.

## Conclusion

A predisposition to HPV-related infections and cancers is common in conditions with immunodeficiency. There seems to be a need for clear guidelines for early diagnosis and management of these immunocompromised conditions of unknown etiology. Gynecologists can encounter these conditions such as in this case presented here. Therefore, persistent, recurrent HPV lesions or their rapid malignant transformations may demand further investigation, especially for conditions or syndromes that affect the immune system.
